# Hospitalization for heart failure incidence according to the transition in metabolic health and obesity status: a nationwide population-based study

**DOI:** 10.1186/s12933-020-01051-2

**Published:** 2020-06-13

**Authors:** You-Bin Lee, Da Hye Kim, Seon Mee Kim, Nan Hee Kim, Kyung Mook Choi, Sei Hyun Baik, Yong Gyu Park, Kyungdo Han, Hye Jin Yoo

**Affiliations:** 1 Division of Endocrinology and Metabolism, Department of Internal Medicine, Korea University College of Medicine, 148 Gurodong-ro, Guro-gu, Seoul, 08308 Republic of Korea; 2 Department of Biostatistics, College of Medicine, The Catholic University of Korea, 222 Banpo-daero Seocho-gu, Seoul, 06591 Republic of Korea; 3 Department of Family Medicine, Korea University College of Medicine, Seoul, Republic of Korea

**Keywords:** Obesity, Metabolic syndrome, Metabolic health, Heart failure

## Abstract

**Background:**

We aimed to investigate the hazard of hospitalization for heart failure (hHF) according to the transitions in metabolic health and obesity status.

**Methods:**

The Korean National Health Insurance Service datasets from 2002 to 2017 were used for this nationwide, longitudinal, population-based study. The hazard of hHF was analyzed according to the eight groups stratified by stability in metabolic health and transition in obesity status among initially metabolically healthy adults who underwent two cycles of health examinations in 2009–2010 and 2013–2014 (N = 7,148,763).

**Results:**

During two examinations, 48.43% of the initially metabolically healthy obese (MHO) individuals and 20.94% of the initially metabolically healthy non-obese (MHNO) individuals showed changes in their metabolic health and obesity status. During a mean follow-up of 3.70 years, 3151 individuals were hospitalized for HF. When stable MHNO individuals were set as the reference, transition to metabolically unhealthy phenotype was associated with an increased hazard of hHF; the hazard ratio (HR) and 95% confidence interval (CI) in the individuals who transformed from MHO to metabolically unhealthy non-obese was 2.033 (1.579–2.616). The constant MHO group had a 17.3% increased hazard of hHF compared with the stable MHNO group [HR (95% CI) 1.173 (1.039–1.325)]. Individuals who shifted from MHO to MHNO showed a 34.3% lower hazard of hHF compared with those who maintained the MHO category [HR (95% CI) 0.657 (0.508–0.849)].

**Conclusion:**

Dynamic changes in metabolic health and obesity status were observed during a relatively short interval of 3–5 years. Loss of metabolic health was significantly associated with an increased hazard of hHF. Even if metabolic health was maintained, persistent obesity remained as a risk factor for hHF, and transition from MHO to MHNO had a protective effect against hHF. Therefore, the prevention and control of obesity while maintaining metabolic health would be crucial in preventing hHF.

## Background

Heart failure (HF) is a complex clinical syndrome that results from any structural or functional impairment in ventricular filling and ejection of blood [1]. HF is a critical public health problem and is the most common cause of hospital admissions in patients aged 65 years or older [2]. People with HF experience a high burden of debilitating symptoms, which limit their daily activities [3, 4]. Due to the limitation in methods to reverse and cure HF, it is important to determine the risk factors for HF and identify individuals that are at an increased risk of developing HF, to establish an effective preventive strategy.

Obesity is a well-known risk factor for incident HF [5–7]. Among 5881 patients in the Framingham Heart Study, HF risk increased by 5% in men and 7% in women according to each single unit increase in body mass index (BMI) after adjusting other known risk factors [5]. Most of the studies that investigated the association of obesity and overweight with HF used BMI to identify overweight and obese patients because of its practicality, but the reliability of BMI as an accurate measure of adiposity is still under debate [8].

Due to the limitations of BMI, which cannot distinguish fat mass and lean mass [6, 9], recent evidences support that considering the presence or absence of combined metabolic abnormality in addition to obesity defined on the basis of BMI can more effectively stratify the prognostic implication of obesity as a risk factor of cardiovascular diseases (CVDs) including HF [10–13]. As a result, concepts of unique obesity sub-phenotypes, including metabolically healthy obesity (MHO) and metabolically unhealthy non-obesity (MUNO) have emerged. In particular, with regard to the risk of HF, Voulgari et al. [14] reported that MHO is associated with a lower risk of HF than is MUNO. However, these obesity sub-phenotypes defined by the status of metabolic health and obesity change dynamically over time [10, 15–19]. Soriguer et al. [18] reported that 41.9% of MHO individuals became metabolically unhealthy during the 6-year follow-up, suggesting that obesity sub-phenotypes should account for the passage of time. Although a few studies tried to reflect the effect of dynamic transitions in obesity sub-phenotypes on the risk of CVD [11, 15–17], most of these studies focused on coronary artery disease, stroke, and/or a composite outcome of multiple CVDs combined with HF rather than HF itself.

To establish an appropriate preventive strategy for HF with regard to obesity and metabolic health, we compared the hazards of incident hospitalization for heart failure (hHF) according to the transitions in obesity sub-phenotypes during the two cycles of health examinations using the Korean National Health Insurance Service (KNHIS) database.

## Methods

### Data sources

We used the KNHIS datasets of claims and preventive health examinations from January 2002 to December 2017 for this study. The KNHIS covers all residents in Korea as a single-insurer organization operated by the Korean government. The KNHIS operates two major programs to offer universal coverage to all residents of Korea: National Health Insurance (NHI) encompassing approximately 97% of the population and Medical Aid (MA) covering the remaining 3% of the population [20]. Since 2006, data of MA beneficiaries have been incorporated into a single KNHIS dataset [20]. Anonymous identification numbers, demographics, monthly income, primary and secondary diagnoses classified according to the International Classification of Diseases-10th Revision (ICD-10), prescriptions, procedures, and dates of hospital visits and hospitalizations of all residents of Korea were incorporated into the KNHIS claims datasets. In addition, the KNHIS promotes standardized preventive health examinations at least every 2 years by actively operating a national health screening program. Demographic data; smoking history; alcohol consumption; physical activity; anthropometric measurements, such as height, weight, waist circumference (WC), and blood pressure (BP); and laboratory data, including liver function tests, estimated glomerular filtration rate (eGFR), lipid profiles, and fasting plasma glucose level, are the components of these standardized health examination. The examination results were assembled into the datasets of preventive health examinations, the largest-scale, nationwide cohort database with laboratory information in Korea. Previous studies have provided details on this database [20–22]. The Institutional Review Board (IRB) of Korea University approved this study (IRB file number 2019GR0329). An informed consent exemption was granted by the IRB because the KNHIS provided the researchers with anonymous, de-identified data only.

### Study Cohort, outcomes, and follow-up

**Fig. 1:**
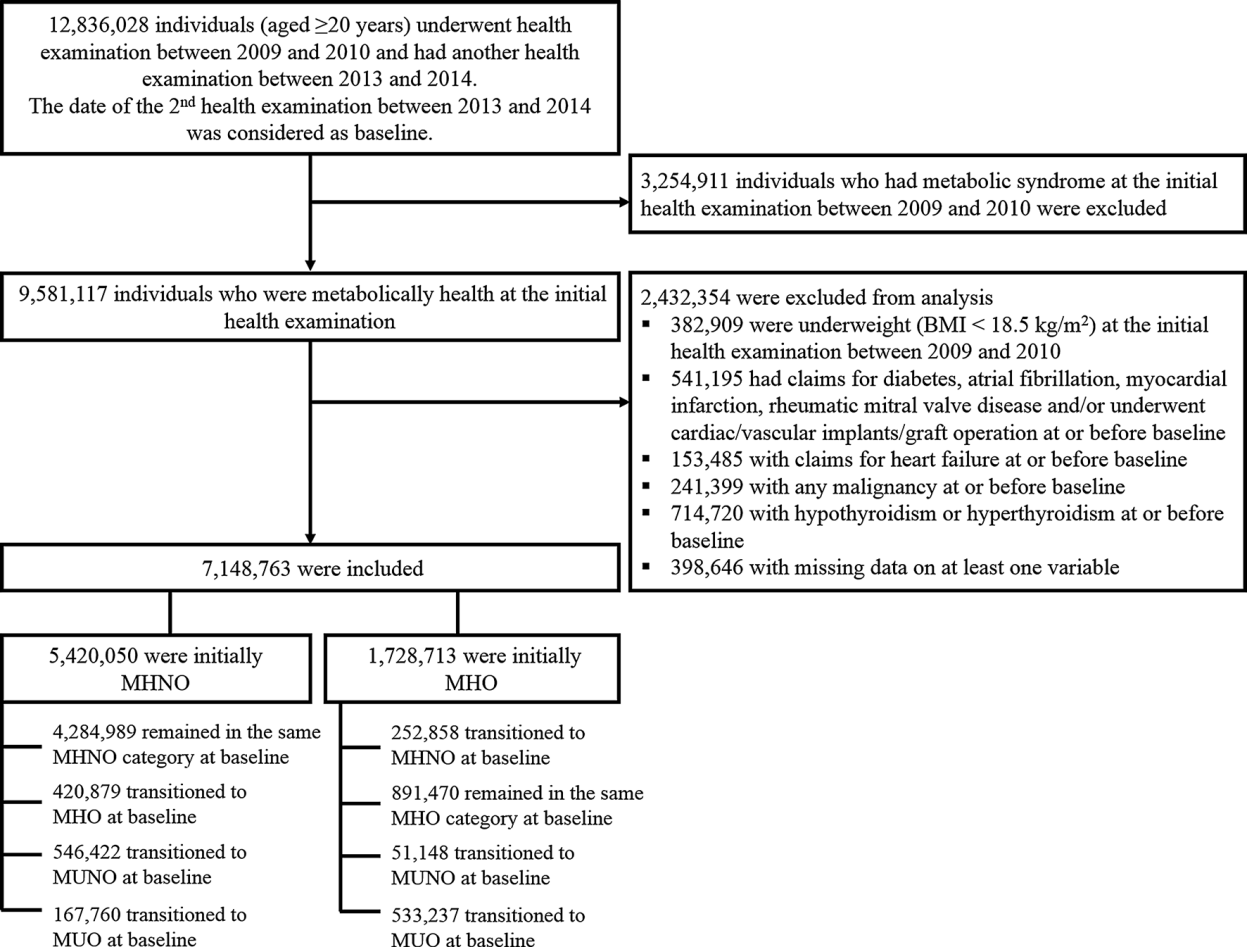
Enrollment, exclusions, and follow-up

In this nationwide, longitudinal, population-based study, we included individuals who met the following criteria: (1) underwent at least one health examination between 2009 and 2010 and were aged ≥ 20 years at the time of this initial health examination, (2) underwent another health examination between 2013 and 2014, and (3) were metabolically healthy (did not have metabolic syndrome [MetS]) at the time of the initial health examination between 2009 and 2010. The time point of the second health examination between 2013 and 2014 was considered as the baseline. Among these, we excluded individuals who were underweight (BMI < 18.5 kg/m^2^) at the time of the first health examination between 2009 and 2010 and those who had, at or before baseline, claims for diabetes mellitus (ICD-10 codes E10–14), atrial fibrillation (AF) (ICD-10 codes I48.0–4 or I48.9), myocardial infarction (MI) (ICD-10 codes I21–22), HF (ICD-10 code I50), and/or rheumatic mitral valve disease (ICD-10 codes I05), and those who had cardiac/vascular implants or grafts (ICD-10 codes Z95) at or before baseline. Furthermore, individuals with any malignancy, hypothyroidism or hyperthyroidism at or before baseline, and missing data in at least one variable were excluded (Fig. 1).

The endpoint was incidence of hHF. The hHF was defined as the first hospitalization under a primary diagnosis of ICD-10 code I50 as reported in previous studies [23–25]. The study population was followed up from baseline (the date of the second health examination between 2013 and 2014) until the date of death, development of hHF, or December 31, 2017, whichever came first.

### Measurements and definitions

Data on smoking history, alcohol consumption, and regular exercise were obtained using questionnaires. Heavy alcohol consumption was considered as an average daily alcohol ingestion of ≥ 30 g. Regular exercise was defined as performance of a high-intensity physical activity (physical activity causing an extreme shortness of breath) for > 20 min per session, ≥ 3 days/week, and/or moderate-intensity physical activity (physical activity accompanied by a substantial shortness of breath) for > 30 min per session, ≥ 5 days/week. Low-income status was regarded as being registered in the MA program for the lowest income population or being part of the lowest one-fifth of the population registered in the NHI, based on the monthly household income. BMI was calculated as body weight in kilograms divided by the height in meters squared (kg/m^2^). Blood tests, including plasma glucose levels and lipid profiles were performed using venous samples obtained after an overnight fast. These health examinations were carried out only in hospitals certified by the KNHIS.

### Definitions of obesity sub-phenotypes

According to the obesity guidelines for the Korean population [26], BMI ≥ 25 kg/m^2^ was used to define obesity. MetS was defined using the harmonized International Diabetes Federation criteria [27], using the cutoffs for abdominal obesity set by the Korean Society for the Study of Obesity (WC ≥ 90 cm for men and ≥ 85 cm for women) [28] (Additional file 1: Table S1). Individuals without MetS were considered metabolically healthy, whereas those with MetS were considered metabolically unhealthy. Obesity sub**-**phenotypes were divided into the following categories according to the presence or absence of obesity and MetS: metabolically healthy non-obesity (MHNO), MHO, MUNO, and metabolically unhealthy obesity (MUO). Since only individuals who were metabolically healthy at the initial health examinations conducted between 2009 and 2010 were included in the current study, all participants were MHNO or MHO at the time of initial examination. At the second examination conducted between 2013 and 2014 (baseline), they transitioned to one of the four obesity sub**-**phenotypes (MHNO, MHO, MUNO, or MUO). All individuals were categorized into eight groups according to the transition in obesity sub**-**phenotypes from the first to the second health examinations (MHNO to MHNO, MHNO to MHO, MHNO to MUNO, MHNO to MUO, MHO to MHNO, MHO to MHO, MHO to MUNO, and MHO to MUO).

### Statistical analyses

The SAS software (Version 9.3, SAS Institute, Cary, NC, USA) was used for all statistical analyses. Two-sided *p*-values < 0.05 were considered significant. The baseline characteristics of the study population were analyzed according to the eight groups stratified by the dynamic changes in obesity sub**-**phenotypes from the first to the second health examinations. Continuous variables with normal distributions are expressed as means ± standard deviations, whereas those with non-normal distributions are presented as geometric means and 95% confidence intervals (CIs). Categorical variables are shown as frequencies and percentages.

The incidence rate of hHF was derived from the number of incident cases divided by the total follow-up duration (person-years). The cumulative incidence rates of hHF according to the eight groups of transition in obesity sub**-**phenotypes were compared using the Kaplan–Meier curves; the differences among the groups were assessed using a log-rank test. A multivariate Cox regression analysis was performed to calculate the hazard ratios (HRs) and 95% CIs for the hHF incidence rates according to the eight groups of transition in obesity sub**-**phenotypes: model 1 was unadjusted, while model 2 was adjusted for age, sex, smoking history, alcohol consumption, regular exercise, and eGFR. Furthermore, additional model (model 3) was constructed which was adjusted for systolic BP, low-density lipoprotein cholesterol (LDL-C), household income in addition to the potential confounders reflected in model 2. These regression models were constructed to include various risk factors for HF as potential confounders, referring to previous reports [14, 29–31]. The proportional hazard assumption of the Cox models was ensured by the Schoenfeld residuals. The HRs (95% CIs) of hHF according to the eight groups of transition in obesity sub**-**phenotypes were calculated in subgroups divided by age (20–44 years, 45–64 years, ≥ 65 years) and sex. In these subgroup analyses, the Cox regression models were adjusted for the same potential confounders reflected in model 2 of the main analysis. The potential effect modification by age group and sex was assessed through this stratified analysis and the *p* for interaction was calculated.

Next, to compare the effect of transition in each MetS component, we selected individuals who satisfied no MetS component at the initial health examination, and then, calculated the HRs (95% CI) of incident hHF according to the transition in each MetS component.

### Sensitivity analyses

We also conducted a sensitivity analysis after excluding individuals who developed hHF within 1 year of follow-up. Furthermore, we performed a sensitivity analysis after excluding individuals with hypertension or dyslipidemia at the first examination. The presence of hypertension and dyslipidemia was defined according to a previous study [32]. In addition, individuals who satisfied two MetS components at the first health examinations were excluded and those with ≤ one MetS component at the initial examinations were selected. Among these selected individuals, another sensitivity analysis after changing the definition of metabolic health to the presence of ≤ one MetS component was conducted.

## Results

### Baseline characteristics and the study population

A total of 7,148,763 individuals were included in the study (Fig. 1). Among them, 5,420,050 were MHNO, while the other 1,728,713 were MHO at the initial health examination conducted between 2009 and 2010. Among the participants who were MHNO at the first examination, 4,284,989 (79.06%) remained in the same MHNO category at the second health examination, while 546,422 (10.08%) and 420,879 (7.77%) individuals transitioned to MUNO and MHO, respectively. The other 167,760 (3.10%) individuals transitioned to MUO. On the contrary, among the participants who were MHO at the first examination, only 891,470 (51.57%) remained in the same MHO phenotype at the second examination, while 533,237 (30.85%) subjects transitioned to MUO at the second examination. The remaining 252,858 (14.63%) and 51,148 (2.96%) participants transitioned to MHNO and MUNO, respectively. The baseline characteristics of the study population according to the eight groups of transition in obesity sub-phenotypes are summarized in Table 1.

**Table 1:** Baseline characteristics of the study population according to the eight groups of transition in obesity subphenotypes

1st examination (2009–2010)	MHNO				MHO				*p*-value
2nd examination (2013–2014)	MHNO	MHO	MUNO	MUO	MHNO	MHO	MUNO	MUO	
*n*	4284989	420,879	546,422	167,760	252,858	891,470	51,148	533,237	
Age (years)	47.95 ± 12.27	44.50 ± 11.64	56.82 ± 11.64	50.33 ± 12.63	49.94 ± 11.83	47.53 ± 11.41	57.38 ± 11.44	50.61 ± 12.09	< 0.0001
Men [n (%)]	2,197,889 (51.29)	278,534 (66.18)	281,416 (51.50)	103,984 (61.98)	165,083 (65.29)	628,772 (70.53)	28,823 (56.35)	357,033 (66.96)	< 0.0001
Current smoker [n (%)]	931,072 (21.73)	110,355 (26.22)	115,043 (21.05)	42,510 (25.34)	63,074 (24.94)	239,997 (26.92)	11,126 (21.75)	146,928 (27.55)	< 0.0001
Heavy alcohol consumption [n (%)]	210,834 (4.92)	30,283 (7.20)	33,326 (6.10)	13,710 (8.17)	16,638 (6.58)	67,853 (7.61)	3411 (6.67)	48,463 (9.09)	< 0.0001
Regular exercise [n (%)]	2,541,420 (59.31)	260,190 (61.82)	293,397 (53.69)	93,740 (55.88)	161,106 (63.71)	570,158 (63.96)	27,892 (54.53)	312,759 (58.65)	< 0.0001
Low-income level [n (%)]	837,078 (19.54)	75,366 (17.91)	125,579 (22.98)	33,867 (20.19)	51,276 (20.28)	164,795 (18.49)	11,868 (23.20)	104,572 (19.61)	< 0.0001
Body weight (kg)	59.49 ± 8.37	72.04 ± 8.25	60.46 ± 8.42	71.68 ± 9.25	65.77 ± 7.76	75.45 ± 9.61	64.27 ± 7.95	77.32 ± 11.25	< 0.0001
Body mass index (kg/m^2^)	21.98 ± 1.72	25.94 ± 0.99	22.86 ± 1.50	26.16 ± 1.16	24.01 ± 0.95	27.22 ± 1.87	24.22 ± 0.82	28.06 ± 2.27	< 0.0001
Waist circumference (cm)	75.86 ± 6.68	84.32 ± 5.64	80.51 ± 6.32	87.35 ± 5.65	80.61 ± 5.66	87.13 ± 6.40	83.69 ± 5.58	90.81 ± 6.61	< 0.0001
Systolic BP (mmHg)	117.44 ± 13.06	121.08 ± 12.19	128.34 ± 13.71	129.13 ± 13.07	120.18 ± 12.82	122.39 ± 12.33	128.46 ± 13.50	129.93 ± 12.95	< 0.0001
Diastolic BP (mmHg)	73.52 ± 9.09	76.01 ± 8.80	79.50 ± 9.49	80.72 ± 9.36	75.20 ± 9.03	76.86 ± 8.86	79.58 ± 9.43	81.36 ± 9.41	< 0.0001
Fasting plasma glucose (mg/dl)	92.33 ± 11.81	93.34 ± 11.25	102.51 ± 16.84	102.15 ± 15.58	93.86 ± 14.94	93.84 ± 12.14	103.49 ± 22.04	102.76 ± 17.75	< 0.0001
Total cholesterol (mg/dl)	193.65 ± 33.13	200.91 ± 33.71	205.38 ± 41.23	207.42 ± 38.75	195.26 ± 33.09	200.79 ± 32.87	202.51 ± 40.57	206.38 ± 37.89	< 0.0001
Triglyceride (mg/dl)	90.83 (90.79-90.88)	111.53 (111.37-111.69)	146.30 (146.10-146.49)	164.24 (163.88-164.61)	94.74 (94.56-94.91)	109.10 (109.00-109.21)	139.24 (138.64-139.84)	157.73 (157.54-157.93)	< 0.0001
HDL-C (mg/dl)	58.84 ± 14.06	54.54 ± 12.66	51.44 ± 14.10	48.87 ± 12.56	56.70 ± 13.59	53.95 ± 12.20	51.14 ± 13.68	48.95 ± 12.19	< 0.0001
LDL-C (mg/dl)	114.72 ± 33.77	121.79 ± 33.90	121.53 ± 40.13	122.88 ± 38.46	117.63 ± 33.02	123.03 ± 33.50	120.39 ± 38.89	123.11 ± 37.63	< 0.0001
eGFR (ml/min/1.73 m^2^)	94.99 ± 52.76	96.06 ± 65.89	89.45 ± 45.04	91.67 ± 53.09	93.21 ± 54.28	93.34 ± 59.90	88.94 ± 47.30	91.17 ± 55.65	< 0.0001
ALT (U/l)	20.65 ± 14.00	28.31 ± 20.48	25.52 ± 16.85	33.28 ± 24.19	22.44 ± 14.75	29.07 ± 20.46	25.63 ± 16.70	35.15 ± 25.43	< 0.0001
AST (U/l)	23.46 ± 10.89	25.48 ± 12.04	26.56 ± 13.85	28.12 ± 14.04	24.13 ± 11.49	26.00 ± 12.17	26.17 ± 13.54	28.77 ± 14.43	< 0.0001
Hypertension [n (%)]	466,017 (10.88)	53,477 (12.71)	246,919 (45.19)	66,006 (39.35)	40,418 (15.98)	151,788 (17.03)	25,130 (49.13)	230,313 (43.19)	< 0.0001
Dyslipidemia [n (%)]	434,994 (10.15)	55,018 (13.07)	267,255 (48.91)	61,665 (36.76)	26,844 (10.62)	110,729 (12.42)	23,703 (46.34)	185,106 (34.71)	< 0.0001

Values are presented as number (%), mean ± standard deviation, or geometric mean (95% confidence interval)

*MHNO* metabolically healthy non-obesity, *MHO* metabolically healthy obesity, *MUNO* metabolically unhealthy non-obesity, *MUO* metabolically unhealthy obesity, *BP* blood pressure, *HDL*-*C* high-density lipoprotein cholesterol, *LDL*-*C* low-density lipoprotein cholesterol, *eGFR* estimated glomerular filtration rate, *ALT* alanine aminotransferase, *AST* aspartate aminotransferase

### Transition in obesity sub-phenotypes and incident hHF

**Fig. 2:**
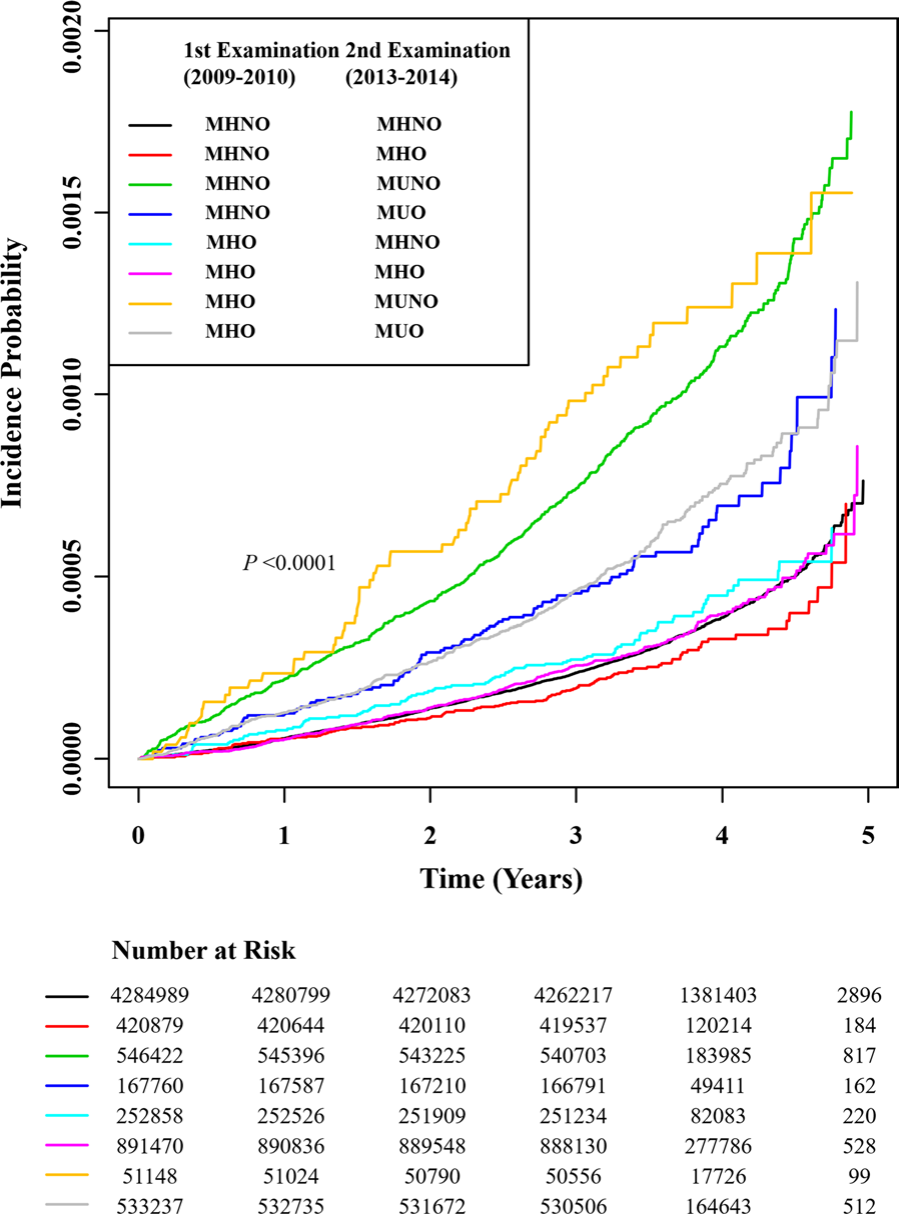
Cumulative incidence of hospitalization for heart failure according to the eight groups stratified by the stability in metabolic health and obesity status. *MHNO* metabolically health non-obesity, *MHO* metabolically healthy obesity, *MUNO* metabolically unhealthy non-obesity, *MUO* metabolically unhealthy obesity

During a mean follow-up of 3.70 ± 0.56 years (26,423,917.95 person-years of follow-up), 3151 participants were hospitalized for HF. The cumulative incidence of hHF is presented according to the eight groups of transition in obesity sub-phenotypes using the Kaplan–Meier curves (Fig. 2). Although all the p-values of pairwise comparisons were statistically significant, the incidence rate of hHF was definitely higher in individuals who had shifted to metabolically unhealthy category at the second examination than in those who consistently maintained metabolic health during the two health examinations (Additional file 2: Fig. S1). The HRs (95% CIs) for hHF incidence were compared according to the eight groups of transition in obesity sub-phenotypes (Table 2). When the individuals who continuously maintained the MHNO phenotype (MHNO to MHNO) were set as the reference group, the MHO to MUNO, MHNO to MUNO, MHO to MUO and MHNO to MUO transition groups had a significant increase in the hazard of hHF [HRs (95% CIs) 2.033 (1.579–2.616), 1.803 (1.637–1.986), 1.677 (1.493–1.884), and 1.548 (1.269–1.887), respectively] in model 2. Furthermore, the hazard of incident hHF was higher in people who remained in the same MHO category than in those who remained in the MHNO category demonstrating a HR (95% CI) of 1.173 (1.039–1.325) in model 2. Conversely, the HR (95% CIs) for hHF in the MHO to MHNO group compared with the MHNO to MHNO group was 0.610 (0.491–0.758) in model 2. Further adjustment for systolic BP, LDL-C, and household income in model 3 also demonstrated consistent results although the strength of association was partially attenuated.

**Table 2:** Hazard ratios and 95% confidence intervals for the incidence of hospitalization for heart failure according to the eight groups of transition in obesity sub-phenotypes

1st examination (2009-2010)	2nd examination (2013-2014)	*n*	Events (n)	Follow-up duration (person-years)	Incidence rate (per 1000 person-years)	Hazard ratios (95% confidence intervals)		
						Model 1	Model 2	Model 3
MHNO	MHNO	4,284,989	1511	15,849,397.29	0.09533	1 (ref.)	1 (ref.)	1 (ref.)
	MHO	420,879	120	1,535,416.61	0.07815	0.831 (0.690, 1.001)	1.116 (0.926, 1.345)	1.096 (0.909, 1.321)
	MUNO	546,422	581	2,049,295.01	0.28351	2.916 (2.649, 3.209)	1.803 (1.637, 1.986)	1.481 (1.341, 1.635)
	MUO	167,760	105	615,952.83	0.17047	1.792 (1.470, 2.184)	1.548 (1.269, 1.887)	1.283 (1.051, 1.566)
MHO	MHNO	252,858	99	936,371.35	0.10573	1.108 (0.904, 1.357)	0.610 (0.491, 0.758)	0.665 (0.537,0.824)
	MHO	891,470	318	3,280,816.52	0.09693	1.022 (0.905, 1.153)	1.173 (1.039, 1.325)	1.132 (1.002, 1.280)
	MUNO	51,148	63	192,110.25	0.32794	3.366 (2.616, 4.331)	2.033 (1.579, 2.616)	1.674 (1.299, 2.156)
	MUO	533,237	354	1,964,558.09	0.18019	1.891 (1.684, 2.123)	1.677 (1.493, 1.884)	1.385 (1.230, 1.560)

Model 1: unadjusted

Model 2: adjusted for age, sex, smoking history, alcohol consumption, regular exercise, and eGFR

Model 3: adjusted for model 2 + systolic BP, LDL-C, and household income

*MHNO* metabolically healthy non-obesity, *MHO* metabolically healthy obesity, *MUNO* metabolically unhealthy non-obesity, *MUO* metabolically unhealthy obesity, *BP* blood pressure, *LDL*-*C* low-density lipoprotein cholesterol

**Fig. 3:**
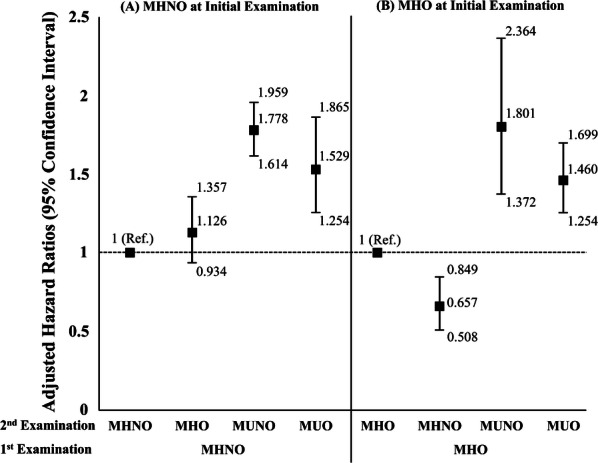
Hazard ratios and 95% confidence intervals for the incidence of hospitalization for heart failure according to the transition in obesity sub-phenotypes among individuals who were MHNO at initial examination (**a**), and among individuals who were MHO at initial examination (**b**). Adjusted for age, sex, smoking history, alcohol consumption, regular exercise, and estimated glomerular filtration rate. *MHNO* metabolically health non-obesity, *MHO* metabolically healthy obesity, *MUNO* metabolically unhealthy non-obesity, *MUO* metabolically unhealthy obesity

Figure 3 summarizes the multivariate-adjusted HRs for incident hHF according to the changes in obesity sub-phenotypes, among initially MHO and MHNO cohorts, respectively. In common, the transition to metabolically unhealthy phenotypes in both cohorts (initially MHNO and MHO cohorts) was associated with a higher hazard of hHF than the maintenance of their initial obesity sub-phenotypes of MHNO or MHO. Among the participants who were initially MHO, transitioning to MHNO was associated with a 34.3% lower hazard of hHF compared with those who remained in the MHO category.

### Subgroup analyses

The hazard of hHF according to the eight groups of transition in obesity sub-phenotypes was assessed in subgroups stratified by age (Additional file 1: Table S2–S4, *p* for interaction in model 2: < 0.0001) and sex (Additional file 1: Table S5–S6, *p* for interaction in model 2: 0.0046). Compared to the stable MHNO group (reference), individuals who had shifted to metabolically unhealthy category at the second examination showed a trend of consistently higher hazard of hHF in all subgroups. However, the increase in the hazard of hHF associated with the transition to metabolically unhealthy category was more prominent in the youngest age group (20-44 years). The higher hazard of incident hHF in the stable MHO category compared with the stable MHNO category was prominent only in individuals aged < 65 years and women. The MHO to MHNO group demonstrated significantly lower hazard of hHF compared to the reference group only in women.

### Transition in metabolic syndrome components and incident hHF

The HRs (95% CIs) for hHF incidence were calculated according to the development of each MetS component among individuals who satisfied no MetS component at the initial health examination (Additional file 1: Table S7). Individuals who developed MetS components of BP, abdominal obesity, high-density lipoprotein cholesterol (HDL-C), and triglyceride demonstrated higher hazards of hHF than those who maintained not to satisfy these components [HRs (95% CIs) 2.811 (2.600–3.040) for BP component; 1.485 (1.365–1.615) for abdominal obesity; 1.212 (1.124–1.308) for HDL-C component; 1.152 (1.069–1.242) for triglyceride component].

### Sensitivity analyses

The main results were consistent when individuals who developed hHF within 1 year of follow-up were excluded (Additional file 1: Table S8). A sensitivity analysis after excluding individuals with hypertension or dyslipidemia at the first examination also demonstrated consistent findings although the trends of increase in the hazard of hHF among stable MHO and the MHNO to MUO groups compared to the reference (stable MHNO) were statistically insignificant (Additional file 1: Table S9). Another sensitivity analysis results after changing the definition of metabolic health to the presence of ≤ one MetS component including only individuals with ≤ one MetS component at the initial examinations are summarized in Additional file 1: Table S10. Also, in this sensitivity analysis, individuals who transitioned to metabolically unhealthy categories had a significantly higher hazard of hHF than the reference group (stable MHNO). Although statistical significance was not secured, individuals who remained in the same MHO category showed a nonsignificant trend of increase in the hazard of hHF compared to the reference group (stable MHNO).

## Discussion

In this large-scale nationwide longitudinal study, we characterized the dynamic nature of the metabolic health status and obesity and demonstrated that transition to metabolically unhealthy phenotypes is associated with an increased hazard of hHF. The individuals who remained in the MHO category were also associated with a 17.3% higher hazard of hHF compared with those who remained in the MHNO category. Furthermore, individuals who transitioned from MHO to MHNO showed a 34.3% lower hazard of hHF compared with those who remained in the MHO category.

In the current study, 48.43% of the participants who were initially MHO and 20.94% of those who were initially MHNO showed changes in their states of metabolic health and obesity during two cycles of health examinations with 3 to 5 years of interval. Intra-individual changes over time in obesity sub-phenotypes should be considered when accounting for the relationship between obesity sub-phenotypes and clinical outcomes. Only a few recent studies [11, 15–17] reflected the transition in obesity sub-phenotypes when evaluating the association between metabolic health and obesity status and cardiovascular outcomes. To the best of our knowledge, this is the first study to examine the relationship between dynamic changes in metabolic health and obesity status and the incidence of hHF.

Obesity causes left atrial structural changes leading to enlargement, increased fibrosis, impaired diastolic function, and accumulation of pericardial fat, ultimately increasing the risk of HF [33]. However, abdominal obesity is more significantly associated with several measures of adverse cardiac functions, independent of BMI [34]. Previously, Lee et al. [35] showed that obesity increased the risk of AF by 20%, whereas metabolic unhealthiness increased the risk of AF risk by 40%. Kim et al. [36] also reported that obesity and diabetes were independently associated with new-onset AF and simultaneously, had synergistic effects on the risk of incident AF. In the current study, transition to metabolically unhealthy phenotypes were associated with approximately 50% or more higher hazard of hHF among individuals who were MHNO or MHO at the initial examination, whereas constantly obese phenotype without metabolic unhealthiness also increased the hazard of hHF by 17% than the stable MHNO phenotype. Among individuals who did not have any MetS components at the initial examination, development of abdominal obesity at the second examination was associated with 48.5% higher hazard of incident hHF than those who did not develop this MetS component at the second examination, demonstrating the second highest excess hazard after the development of BP component among the five components of MetS. Interestingly, the hazard of hHF in the participants who transitioned from MHO to MUNO was as high as in those who transitioned from MHO to MUO. Although the characteristics of these participants (MHO to MUNO), whose weight was reduced as they transitioned to metabolic unhealthiness, could not be clarified in this study, they might have increased proportion of visceral fat as their body composition. Visceral fat is an active endocrine organ producing cytokines and generating a systemic proinflammatory condition that, in turn, promotes CVD [37, 38]. Hyperinsulinemia caused by visceral adipose tissue, the main component of metabolic unhealthiness, induces sympathetic nervous system activation, endothelial dysfunction, and inhibition of nitric oxide synthase [39, 40], all of which contribute to the development of HF. Neeland et al. [41] showed that visceral adipose burden can be used to identify otherwise healthy individuals with greater left ventricular (LV) hypertrophy and concentric remodeling regardless of BMI. Likewise, Abbasi et al. [42] reported that only visceral fat area, but not subcutaneous area, was significantly associated with LV concentricity and LV mass index, a precursor to HF. Therefore, investigation of these distinct adipose tissue depots is essential to better define the mechanisms by which obesity leads to HF.

Although the present study suggests that the loss of metabolic health may be a stronger predictor of hHF with higher priority than the transition in obesity status, consistently MHO individuals showed a mild increase in the hazard of hHF compared with those who continuously maintained the MHNO category. Similarly, Rozenbaum et al. [43] demonstrated that high BMI is associated with increased risk of diastolic dysfunction even in metabolically healthy individuals. In addition, Morkedal et al. [44] suggested that long-lasting obesity had a more detrimental effect on the risk of incident HF than the incident acute MI. In that study, MHO did not confer an excess risk of acute MI, whereas MHO was associated with a higher risk of HF [44]. In the current study, individuals who transitioned from MHO to MHNO had a lower hazard of hHF than did those who remained in the MHO category. This finding indicates that restoring the non-obese state from obesity while maintaining metabolic health may have a protective effect against incident hHF. In a previous study from a Swedish nationwide registry of obese people, people who underwent gastric bypass surgery lost 18.8 kg more weight after 1 year and 22.6 kg more after 2 years on average and had a lower hazard of incident HF with an HR of 0.54 (95% CI 0.36–0.82) than did people who were managed through lifestyle modification only [45]. Treatment modalities proven to have beneficial effects both on metabolic health and obesity including bariatric surgery [46, 47], sodium glucose cotransporter 2 inhibitors [48, 49] and glucagon-like polypeptide-1 receptor agonists [50, 51] might also be a preventive strategy for incident hHF.

In stratified analyses according to age and sex, the excess risk of hHF in individuals who transitioned to metabolically unhealthy category was more prominent in the younger age group. The excess hazard of incident hHF in the stable MHO category compared to the stable MHNO was prominent only in individuals aged < 65 years and women. Similarly, in previous studies, the relationship between diabetes and increased risk of HF was much stronger in individuals of younger age (≤ 65 years [52] or < 55 years [53]). Furthermore, in a previous report [12], the excess risk of HF in overweight individuals without metabolic abnormalities differed significantly by age and sex demonstrating a stronger positive association in individuals aged < 65 years than in those aged ≥ 65 years, and in females than in males. Although the exact mechanisms cannot be clarified, these consistent findings suggest the possible vulnerability of these sub-populations to metabolic deterioration and/or persistent obesity. Moreover, in respect to a more prominent association in younger individuals, from a statistical point of view, with increasing age more individuals are at risk for the hHF incidence in general, possibly attenuating the excess effect of metabolic derangement or persistent MHO. For women, more prominent protective effect of MHO to MHNO category against the incident hHF and the more prominent association between stable MHO and the excess hazard of hHF may be originated from the dependency of BMI (which was used to define obesity in the current study) [54] on sex as a measure to reflect body fat mass. Previous studies have consistently reported that, for the same BMI, women have greater amounts of fat mass than men throughout the entire adult life [55–57].

Several limitations of this study should be acknowledged. First, because of the observational nature of this study, it is inevitably limited to clarify the causal relationships. However, to minimize the possible reverse causality effect, individuals with claims for HF at or before baseline were excluded, and the transition in obesity sub-phenotype was assessed prior to the baseline examination. Furthermore, a sensitivity analysis after excluding people who developed hHF within 1 year of follow-up demonstrated consistent results. Second, considering that the highest mean BMI of the participants according to the eight groups of transition in obesity sub-phenotypes was 28.06 kg/m^2^, our results may not be generalizable to people with more severe obesity. Third, the initiation and discontinuation of drugs that can potentially affect the incidence of hHF, including renin-angiotensin system inhibitors, beta blockers, and statins were not reflected as potential confounders. However, since our study was conducted after excluding individuals with baseline HF, MI, AF, rheumatic mitral valve disease, cardiac/vascular graft/implants, and/or diabetes, effects of transition in obesity and metabolic health status on the incidence of hHF was evaluated in a relatively low CVD risk population with primary prevention setting. In this low risk population without previous CVDs, incidence of hHF may be less affected by the medication use than in the population with previous CVDs or high risk of CVD. Fourth, since the outcome of our study was limited to hHF, association between transitions in obesity sub-phenotypes and the incidence of milder HF that do not require hospitalization has not been fully evaluated. Furthermore, due to the unavailability of data, HF subtype according to ejection fraction was not differentiated while a recent study suggests that obesity and related cardiometabolic traits including insulin resistance are more strongly associated with risk of incident HF with preserved versus reduced ejection fraction (HFpEF versus HFrEF) [58]. Despite these limitations, our study has major strengths. We used a validated nationwide cohort database representing the Korean population provided by the Korean government. The KNHIS database, which was used in the current study, includes the lifestyle and laboratory data of a large sample, enabling adjustment for diverse confounding factors.

## Conclusions

In this large population-based study, loss of metabolic health was associated with an increased hazard of hHF. In addition, even when metabolic health was maintained, persistent obesity was associated with a higher hazard of hHF than was stable MHNO category. Transition from MHO to MHNO may have a protective effect against future hHF. Therefore, obesity as a predictor of hHF should be considered, accounting for its dynamic changes as well as the transition in metabolic health status. As a clinical implication, the development of obesity should be prevented, and the prevalent obesity should be managed actively while strictly maintaining metabolic health for the prevention of hHF.

## Supplementary information

**Additional file 1.** Additional figure legend and tables.

**Additional file 2.** Additional figure S1.

## Data Availability

The data that support the findings of this study are available from the Korean National Health Insurance Service (KNHIS), but restrictions apply to their availability, which were used under license for the current study and so are not publicly available. However, data are available from the authors upon reasonable request and with permission of the KNHIS.

## Abbreviations

AF: Atrial fibrillation
BMI: Body mass index
BP: Blood pressure
CI: Confidence interval
CVD: Cardiovascular disease
eGFR: Estimated glomerular filtration rate
HDL-C: High-density lipoprotein cholesterol
HF: Heart failure
hHF: Hospitalization for heart failure
HR: Hazard ratio
ICD-10: International Classification of Diseases-10th Revision
IRB: Institutional Review Board
KNHIS: Korean National Health Insurance Service
LDL-C: Low-density lipoprotein cholesterol
LV: Left ventricular
MA: Medical Aid
MetS: Metabolic syndrome
MHNO: Metabolically healthy non-obesity
MHO: Metabolically healthy obesity
MI: Myocardial infarction
MUNO: Metabolically unhealthy non-obesity
MUO: Metabolically unhealthy obesity
NHI: National Health Insurance
WC: Waist circumference
