# Supplementary material for: Hospitalization for heart failure incidence according to the transition in metabolic health and obesity status: a nationwide population-based study
Source: Cardiovasc Diabetol. 2020 Jun 13;19:77. doi: 10.1186/s12933-020-01051-2 (PMC7293788; doi:10.1186/s12933-020-01051-2)
Supplement: Supplementary file 1 — Additional file 1. Additional figure legend and tables. [file 12933_2020_1051_MOESM1_ESM.docx]

**Hospitalization for heart failure incidence according to the transition in metabolic health and obesity status: A nationwide population-based study**

You-Bin Lee, MD^1^; Da Hye Kim, BS^2^; Seon Mee Kim, MD, PhD^3^; Nan Hee Kim, MD, PhD^1^; Kyung Mook Choi, MD, PhD^1^; Sei Hyun Baik, MD, PhD^1^; Yong Gyu Park, PhD^2^; Kyungdo Han, PhD^2*^; and Hye Jin Yoo, MD, PhD^1*^

^1^Division of Endocrinology and Metabolism, Department of Internal Medicine, Korea University College of Medicine, Seoul, Republic of Korea

^2^Department of Biostatistics, College of Medicine, The Catholic University of Korea, Seoul, Republic of Korea

^3^Department of Family Medicine, Korea University College of Medicine, Seoul, Republic of Korea

*** Address for correspondence**

Hye Jin Yoo, MD, PhD

Division of Endocrinology and Metabolism, Department of Internal Medicine, Korea University College of Medicine

148 Gurodong-ro, Guro-gu, Seoul 08308, Republic of Korea

Phone: +82-2-2626-3045

Fax: +82-2-2626-1096

E-mail: deisy21@naver.com

Kyungdo Han, PhD

Department of Biostatistics, College of Medicine, The Catholic University of Korea

222 Banpo-daero Seocho-gu, Seoul, 06591, Republic of Korea

Phone: +82-2-2258-7230

Fax: +82-2-532-6537

E-mail: hkd917@naver.com

***Abbreviated Title:*** Obesity sub-phenotype changes and heart failure

**Supplementary figure legends**

**Fig. S1.** Cumulative incidence of hospitalization for heart failure according to the eight groups stratified by the stability in metabolic health and obesity status. Pairwise comparisons of each curve with the curve of the reference (stable MHNO) group was additionally provided using Bonferroni correction. The dashed lines represent the 95% confidence interval. As a result, all the p-values of pairwise comparisons were statistically significant. Abbreviations: MHNO, metabolically health non-obesity; MHO, metabolically healthy obesity, MUNO, metabolically unhealthy non-obesity; MUO, metabolically unhealthy obesity

**Table S1** Definition of metabolic syndrome

| Harmonized International Diabetes Federation criteria for metabolic syndrome: ≥3 of the following components |
| --- |
| - Triglyceride level ≥150 mg/dl or medication use - HDL-C <40 mg/dl in men and <50 mg/dl in women or medication use - SBP ≥130 mm Hg or DBP ≥85 mm Hg or BP medication use - Fasting glucose ≥100 mg/dl† - Abdominal obesity: waist circumference: ≥90 cm in men, ≥85 cm in women according to the Korean Society for the Study of Obesity |

†Individuals who had claims for diabetes mellitus at or before baseline were excluded from analysis.

Abbreviations: HDL-C, high-density lipoprotein cholesterol; SBP, systolic blood pressure; DBP, diastolic blood pressure; BP, blood pressure.

**Table S2** Hazard ratios and 95% confidence intervals for the incidence of hospitalization for heart failure according to the eight groups of transition in obesity sub-phenotypes, subgroup analysis among individuals aged 20-44 years

| 1^st^ examination (2009-2010) | 2^nd^ examination (2013-2014) | *n* | Events (n) | Follow-up duration (person-years) | Incidence rate (per 1000 person-years) | Hazard ratios (95% confidence intervals) | |  |
| --- | --- | --- | --- | --- | --- | --- | --- | --- |
|  |  |  |  |  |  | Model 1 | Model 2 | |
| MHNO | MHNO | 1807853 | 66 | 6604681.00 | 0.00999 | 1(ref.) | 1(ref.) | |
|  | MHO | 228859 | 14 | 825508.24 | 0.01696 | 1.708 (0.959, 3.041) | 1.758 (0.983, 3.143) | |
|  | MUNO | 84004 | 19 | 303233.12 | 0.06266 | 6.297 (3.780, 10.490) | 5.341 (3.177, 8.980) | |
|  | MUO | 58900 | 7 | 210240.82 | 0.03330 | 3.365 (1.544, 7.335) | 3.148 (1.437, 6.895) | |
| MHO | MHNO | 90648 | 6 | 330421.65 | 0.01816 | 1.819 (0.789, 4.196) | 1.687 (0.729, 3.905) | |
|  | MHO | 392236 | 33 | 1424424.77 | 0.02317 | 2.325 (1.531, 3.531) | 2.205 (1.437, 3.385) | |
|  | MUNO | 7483 | 2 | 27078.88 | 0.07386 | 7.404 (1.814, 30.228) | 5.936 (1.446, 24.380) | |
|  | MUO | 183933 | 30 | 660601.08 | 0.04541 | 4.578 (2.973, 7.049) | 4.065 (2.602, 6.350) | |

Model 1: unadjusted.

Model 2: adjusted for age, sex, smoking history, alcohol consumption, regular exercise, and eGFR.

Abbreviations: MHNO, metabolically healthy non-obesity; MHO, metabolically healthy obesity; MUNO, metabolically unhealthy non-obesity; MUO, metabolically unhealthy obesity.

**Table S3** Hazard ratios and 95% confidence intervals for the incidence of hospitalization for heart failure according to the eight groups of transition in obesity sub-phenotypes, subgroup analysis among individuals aged 45-64 years

| 1^st^ examination (2009-2010) | 2^nd^ examination (2013-2014) | *n* | Events (n) | Follow-up duration (person-years) | Incidence rate (per 1000 person-years) | Hazard ratios (95% confidence intervals) | |  |
| --- | --- | --- | --- | --- | --- | --- | --- | --- |
|  |  |  |  |  |  | Model 1 | Model 2 | |
| MHNO | MHNO | 2051861 | 381 | 7596591.68 | 0.05015 | 1(ref.) | 1(ref.) | |
|  | MHO | 167431 | 46 | 614343.64 | 0.07488 | 1.499 (1.104, 2.035) | 1.547 (1.139, 2.102) | |
|  | MUNO | 324482 | 89 | 1209300.22 | 0.07360 | 1.463 (1.161, 1.842) | 1.222 (0.969, 1.541) | |
|  | MUO | 85513 | 35 | 314911.35 | 0.11114 | 2.221 (1.571, 3.139) | 1.994 (1.410, 2.820) | |
| MHO | MHNO | 132010 | 26 | 489238.08 | 0.05314 | 1.059 (0.712, 1.576) | 0.991 (0.666, 1.475) | |
|  | MHO | 426364 | 125 | 1573468.05 | 0.07944 | 1.586 (1.296, 1.941) | 1.556 (1.270, 1.905) | |
|  | MUNO | 29769 | 10 | 111088.73 | 0.09002 | 1.788 (0.954, 3.350) | 1.461 (0.779, 2.738) | |
|  | MUO | 276217 | 91 | 1019556.88 | 0.08925 | 1.782 (1.417, 2.239) | 1.599 (1.271, 2.011) | |

Model 1: unadjusted.

Model 2: adjusted for age, sex, smoking history, alcohol consumption, regular exercise, and eGFR.

Abbreviations: MHNO, metabolically healthy non-obesity; MHO, metabolically healthy obesity; MUNO, metabolically unhealthy non-obesity; MUO, metabolically unhealthy obesity.

**Table S4** Hazard ratios and 95% confidence intervals for the incidence of hospitalization for heart failure according to the eight groups of transition in obesity sub-phenotypes, subgroup analysis among individuals aged ≥65years

| 1^st^ examination (2009-2010) | 2^nd^ examination (2013-2014) | *n* | Events (n) | Follow-up duration (person-years) | Incidence rate (per 1000 person-years) | Hazard ratios (95% confidence intervals) | |  |
| --- | --- | --- | --- | --- | --- | --- | --- | --- |
|  |  |  |  |  |  | Model 1 | Model 2 | |
| MHNO | MHNO | 425275 | 1064 | 1648124.61 | 0.64558 | 1(ref.) | 1(ref.) | |
|  | MHO | 24589 | 60 | 95564.73 | 0.62785 | 0.972 (0.749, 1.260) | 1.062 (0.818, 1.377) | |
|  | MUNO | 137936 | 473 | 536761.67 | 0.88121 | 1.362 (1.222, 1.517) | 1.345 (1.205, 1.501) | |
|  | MUO | 23347 | 63 | 90800.66 | 0.69383 | 1.073 (0.832, 1.384) | 1.112 (0.862, 1.434) | |
| MHO | MHNO | 30200 | 67 | 116711.62 | 0.57406 | 0.892 (0.697, 1.141) | 0.743 (0.575, 0.961) | |
|  | MHO | 72870 | 160 | 282923.69 | 0.56552 | 0.878 (0.743, 1.036) | 0.993 (0.840, 1.173) | |
|  | MUNO | 13896 | 51 | 53942.64 | 0.94545 | 1.463 (1.105, 1.938) | 1.470 (1.109, 1.948) | |
|  | MUO | 73087 | 233 | 284400.13 | 0.81927 | 1.268 (1.101, 1.462) | 1.369 (1.186, 1.580) | |

Model 1: unadjusted.

Model 2: adjusted for age, sex, smoking history, alcohol consumption, regular exercise, and eGFR.

Abbreviations: MHNO, metabolically healthy non-obesity; MHO, metabolically healthy obesity; MUNO, metabolically unhealthy non-obesity; MUO, metabolically unhealthy obesity.

**Table S5** Hazard ratios and 95% confidence intervals for the incidence of hospitalization for heart failure according to the eight groups of transition in obesity sub-phenotypes among male participants

| 1^st^ examination (2009-2010) | 2^nd^ examination (2013-2014) | *n* | Events (n) | Follow-up duration (person-years) | Incidence rate (per 1000 person-years) | Hazard ratios (95% confidence intervals) | |  |
| --- | --- | --- | --- | --- | --- | --- | --- | --- |
|  |  |  |  |  |  | Model 1 | Model 2 | |
| MHNO | MHNO | 2197889 | 904 | 8057565.52 | 0.11219 | 1(ref.) | 1(ref.) | |
|  | MHO | 278534 | 73 | 1008515.88 | 0.07238 | 0.655 (0.516, 0.831) | 1.045 (0.822, 1.328) | |
|  | MUNO | 281416 | 289 | 1035046.76 | 0.27921 | 2.462 (2.157, 2.811) | 1.823 (1.595, 2.083) | |
|  | MUO | 103984 | 53 | 376239.18 | 0.14087 | 1.268 (0.961, 1.672) | 1.393 (1.055, 1.839) | |
| MHO | MHNO | 165083 | 54 | 606126.63 | 0.08909 | 0.793 (0.603, 1.044) | 0.884 (0.671, 1.164) | |
|  | MHO | 628772 | 195 | 2294057.02 | 0.08500 | 0.763 (0.654, 0.891) | 1.084 (0.926, 1.268) | |
|  | MUNO | 28823 | 30 | 106253.84 | 0.28234 | 2.486 (1.728, 3.576) | 1.902 (1.321, 2.737) | |
|  | MUO | 357033 | 182 | 1295818.59 | 0.14045 | 1.263 (1.077, 1.481) | 1.494 (1.272, 1.755) | |

Model 1: unadjusted.

Model 2: adjusted for age, sex, smoking history, alcohol consumption, regular exercise, and eGFR.

Abbreviations: MHNO, metabolically healthy non-obesity; MHO, metabolically healthy obesity; MUNO, metabolically unhealthy non-obesity; MUO, metabolically unhealthy obesity.

**Table S6** Hazard ratios and 95% confidence intervals for the incidence of hospitalization for heart failure according to the eight groups of transition in obesity sub-phenotypes among female participants

| 1^st^ examination (2009-2010) | 2^nd^ examination (2013-2014) | *n* | Events (n) | Follow-up duration (person-years) | Incidence rate (per 1000 person-years) | Hazard ratios (95% confidence intervals) | |  |
| --- | --- | --- | --- | --- | --- | --- | --- | --- |
|  |  |  |  |  |  | Model 1 | Model 2 | |
| MHNO | MHNO | 2087100 | 607 | 7791831.77 | 0.07790 | 1(ref.) | 1(ref.) | |
|  | MHO | 142345 | 47 | 526900.73 | 0.08920 | 1.151 (0.856, 1.549) | 1.211 (0.900, 1.629) | |
|  | MUNO | 265006 | 292 | 1014248.24 | 0.28790 | 3.603 (3.133, 4.143) | 1.582 (1.371, 1.825) | |
|  | MUO | 63776 | 52 | 239713.65 | 0.21693 | 2.755 (2.076, 3.657) | 1.596 (1.202, 2.120) | |
| MHO | MHNO | 87775 | 45 | 330244.72 | 0.13626 | 1.736 (1.283, 2.350) | 0.031 (0.013, 0.077) | |
|  | MHO | 262698 | 123 | 986759.49 | 0.12465 | 1.588 (1.309, 1.928) | 1.250 (1.030, 1.517) | |
|  | MUNO | 22325 | 33 | 85856.41 | 0.38436 | 4.785 (3.371, 6.793) | 1.894 (1.332, 2.693) | |
|  | MUO | 176204 | 172 | 668739.5 | 0.25720 | 3.241 (2.736, 3.839) | 1.741 (1.468, 2.065) | |

Model 1: unadjusted.

Model 2: adjusted for age, sex, smoking history, alcohol consumption, regular exercise, and eGFR.

Abbreviations: MHNO, metabolically healthy non-obesity; MHO, metabolically healthy obesity; MUNO, metabolically unhealthy non-obesity; MUO, metabolically unhealthy obesity.

**Table S7** Hazard ratios and 95% confidence intervals for the incidence of hospitalization for heart failure according to transition in metabolic syndrome components, including only individuals who satisfied no metabolic syndrome component at the initial health examination

| 1^st^ examination (2009-2010) | 2^nd^ examination (2013-2014) | *n* | Events (n) | Follow-up duration (person-years) | Incidence rate (per 1000 person-years) | Hazard ratios (95% confidence intervals) | |
| --- | --- | --- | --- | --- | --- | --- | --- |
|  |  |  |  |  |  | Model 1 | Model 2 |
| **BP ≥ 130/85 mmHg or medication use** | | | | | | | |
| No | No | 4531407 | 948 | 16741119.43 | 0.05663 | 1(ref.) | 1(ref.) |
|  | Yes | 2617356 | 2203 | 9682798.51 | 0.22752 | 3.986 (3.694, 4.301) | 2.811 (2.600, 3.040) |
| **Triglyceride level ≥ 150 mg/dl or medication use** | | | | | | | |
| No | No | 5093627 | 2084 | 18818400.00 | 0.11074 | 1(ref.) | 1(ref.) |
|  | Yes | 2055136 | 1067 | 7605517.94 | 0.14029 | 1.264 (1.174, 1.361) | 1.152 (1.069, 1.242) |
| **HDL-C <40 mg/dl in men and <50 mg/dl in women or medication use** | | | | | | | |
| No | No | 5572292 | 2082 | 20526198.42 | 0.10143 | 1(ref.) | 1(ref.) |
|  | Yes | 1576471 | 1069 | 5897719.52 | 0.18126 | 1.756 (1.631, 1.890) | 1.212 (1.124, 1.308) |
| **Abdominal obesity^†^** | | | | | | | |
| No | No | 6069648 | 2439 | 22441178.39 | 0.10868 | 1(ref.) | 1(ref.) |
|  | Yes | 1079115 | 712 | 3982739.55 | 0.17877 | 1.643 (1.511, 1.786) | 1.485 (1.365, 1.615) |
| **Fasting glucose ≥100 mg/dl**^‡^ | | | | | | | |
| No | No | 5215508 | 2042 | 19324082.85 | 0.10567 | 1(ref.) | 1(ref.) |
|  | Yes | 1933255 | 1109 | 7099835.10 | 0.15620 | 1.484 (1.379, 1.596) | 1.078 (0.998, 1.164) |

^†^Cut-off values for abdominal obesity: waist circumference: ≥90 cm in men, ≥85 cm in women according to the Korean Society for the Study of Obesity.

^‡^Individuals who had claims for diabetes mellitus at or before baseline were excluded from analysis.

Model 1: unadjusted.

Model 2: adjusted for age, sex, smoking history, alcohol consumption, regular exercise, and eGFR.

Abbreviations: MHNO, metabolically healthy non-obesity; MHO, metabolically healthy obesity; MUNO, metabolically unhealthy non-obesity; MUO, metabolically unhealthy obesity; BP, blood pressure; HDL-C, high-density lipoprotein cholesterol.

**Table S8** Hazard ratios and 95% confidence intervals for the incidence of hospitalization for heart failure according to the eight groups of transition in obesity sub-phenotypes, sensitivity analysis after excluding individuals who developed outcome within one year of follow-up

| 1^st^ examination (2009-2010) | 2^nd^ examination (2013-2014) | *n* | Events (n) | Follow-up duration (person-years) | Incidence rate (per 1000 person-years) | Hazard ratios (95% confidence intervals) | |
| --- | --- | --- | --- | --- | --- | --- | --- |
|  |  |  |  |  |  | Model 1 | Model 2 |
| MHNO | MHNO | 4280781 | 1269 | 11565801.13 | 0.10972 | 1(ref.) | 1(ref.) |
|  | MHO | 420642 | 97 | 1114614.20 | 0.08703 | 0.804 (0.654, 0.989) | 1.080 (0.878, 1.328) |
|  | MUNO | 545391 | 461 | 1503238.37 | 0.30667 | 2.735 (2.458, 3.043) | 1.695 (1.523, 1.888) |
|  | MUO | 167587 | 85 | 448253.21 | 0.18962 | 1.731 (1.390, 2.156) | 1.491 (1.196, 1.858) |
| MHO | MHNO | 252526 | 79 | 683623.13 | 0.11556 | 1.052 (0.838, 1.320) | 0.573 (0.450,0.730) |
|  | MHO | 890832 | 270 | 2389570.87 | 0.11299 | 1.035 (0.908, 1.180) | 1.189 (1.042, 1.358) |
|  | MUNO | 51023 | 51 | 141003.76 | 0.36169 | 3.219 (2.433, 4.258) | 1.949 (1.472, 2.579) |
|  | MUO | 532733 | 286 | 1431500.25 | 0.19979 | 1.820 (1.601, 2.070) | 1.613 (1.418, 1.835) |

Model 1: unadjusted.

Model 2: adjusted for age, sex, smoking history, alcohol consumption, regular exercise, and eGFR.

Abbreviations: MHNO, metabolically healthy non-obesity; MHO, metabolically healthy obesity; MUNO, metabolically unhealthy non-obesity; MUO, metabolically unhealthy obesity.

**Table S9** Hazard ratios and 95% confidence intervals for the incidence of hospitalization for heart failure according to the eight groups of transition in obesity sub-phenotypes, sensitivity analysis after excluding individuals who had hypertension or dyslipidemia at the time of the first health examination between 2009 and 2010

| 1^st^ examination (2009-2010) | 2^nd^ examination (2013-2014) | *n* | Events (n) | Follow-up duration (person-years) | Incidence rate (per 1000 person-years) | Hazard ratios (95% confidence intervals) | |  |
| --- | --- | --- | --- | --- | --- | --- | --- | --- |
|  |  |  |  |  |  | Model 1 | Model 2 | |
| MHNO | MHNO | 3611010 | 875 | 13331230.48 | 0.06564 | 1(ref.) | 1(ref.) | |
|  | MHO | 355904 | 65 | 1295953.82 | 0.05016 | 0.774 (0.602, 0.996) | 1.011 (0.785, 1.302) | |
|  | MUNO | 308202 | 225 | 1149300.74 | 0.19577 | 2.932 (2.532, 3.395) | 1.860 (1.605, 2.155) | |
|  | MUO | 111736 | 35 | 407838.54 | 0.08582 | 1.315 (0.938, 1.844) | 1.192 (0.850, 1.672) | |
| MHO | MHNO | 189550 | 55 | 701023.50 | 0.07846 | 1.193 (0.909, 1.567) | 0.648 (0.483, 0.868) | |
|  | MHO | 689373 | 162 | 2533770.40 | 0.06394 | 0.978 (0.827, 1.156) | 1.090 (0.921, 1.290) | |
|  | MUNO | 26438 | 25 | 99002.68 | 0.25252 | 3.766 (2.531, 5.603) | 2.301 (1.546, 3.426) | |
|  | MUO | 329455 | 147 | 1208162.31 | 0.12167 | 1.859 (1.561, 2.213) | 1.694 (1.421, 2.019) | |

Model 1: unadjusted.

Model 2: adjusted for age, sex, smoking history, alcohol consumption, regular exercise, and eGFR.

Abbreviations: MHNO, metabolically healthy non-obesity; MHO, metabolically healthy obesity; MUNO, metabolically unhealthy non-obesity; MUO, metabolically unhealthy obesity.

**Table S10** Hazard ratios and 95% confidence intervals for the incidence of hospitalization for heart failure according to the eight groups of transition in obesity sub-phenotypes, sensitivity analysis after changing the definition of metabolic health to the presence of ≤one metabolic syndrome component

| 1^st^ examination (2009-2010) | 2^nd^ examination (2013-2014) | *n* | Events (n) | Follow-up duration (person-years) | Incidence rate (per 1000 person-years) | Hazard ratios (95% confidence intervals) | |  |
| --- | --- | --- | --- | --- | --- | --- | --- | --- |
|  |  |  |  |  |  | Model 1 | Model 2 | |
| MHNO | MHNO | 2910975 | 664 | 10746021.17 | 0.06179 | 1(ref.) | 1(ref.) | |
|  | MHO | 212230 | 34 | 773446.14 | 0.04396 | 0.721 (0.511, 1.018) | 1.197 (0.848, 1.691) | |
|  | MUNO | 893767 | 621 | 3320249.42 | 0.18703 | 2.987 (2.677, 3.332) | 1.466 (1.312, 1.637) | |
|  | MUO | 218095 | 85 | 794218.04 | 0.10702 | 1.749 (1.396, 2.192) | 1.641 (1.309, 2.058) | |
| MHO | MHNO | 118337 | 25 | 437066.93 | 0.05720 | 0.926 (0.621, 1.380) | 0.883 (0.592, 1.317) | |
|  | MHO | 306992 | 59 | 1128420.75 | 0.05229 | 0.850 (0.652, 1.110) | 1.132 (0.866, 1.479) | |
|  | MUNO | 51985 | 42 | 193678.16 | 0.21685 | 3.455 (2.529, 4.719) | 1.722 (1.260, 2.353) | |
|  | MUO | 408774 | 159 | 1498869.30 | 0.10608 | 1.722 (1.448, 2.047) | 1.585 (1.332, 1.885) | |

Model 1: unadjusted.

Model 2: adjusted for age, sex, smoking history, alcohol consumption, regular exercise, and eGFR.

Abbreviations: MHNO, metabolically healthy non-obesity; MHO, metabolically healthy obesity; MUNO, metabolically unhealthy non-obesity; MUO, metabolically unhealthy obesity.
