# Supplementary figures and images for: Hospitalization for heart failure incidence according to the transition in metabolic health and obesity status: a nationwide population-based study
Source: Cardiovasc Diabetol. 2020 Jun 13;19:77. doi: 10.1186/s12933-020-01051-2 (PMC7293788; doi:10.1186/s12933-020-01051-2)

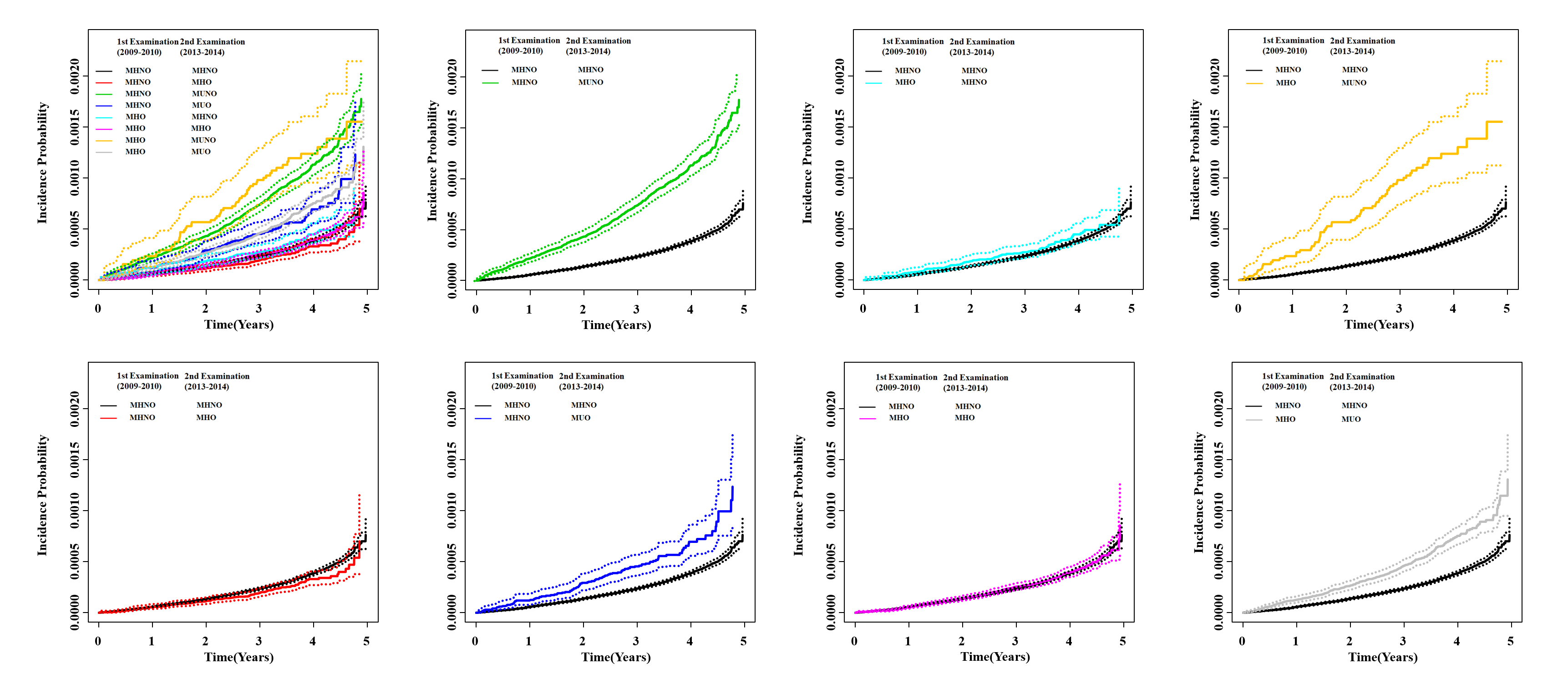

Supplement: Supplementary file 2 — Additional file 2. Additional figure S1. [file 12933_2020_1051_MOESM2_ESM.tif]
